# Differential Expression of *SMAD* Genes and *S1PR1* on Circulating CD4+ T Cells in Multiple Sclerosis and Crohn’s Disease

**DOI:** 10.3390/ijms21020676

**Published:** 2020-01-20

**Authors:** Judith Abarca-Zabalía, Ma Isabel García, Alberto Lozano Ros, Ignacio Marín-Jiménez, Maria L. Martínez-Ginés, Beatriz López-Cauce, María L. Martín-Barbero, Sara Salvador-Martín, María Sanjurjo-Saez, Jose M. García-Domínguez, Luis A. López Fernández

**Affiliations:** 1 Servicio de Farmacia, Instituto de Investigación Sanitaria Gregorio Marañón, Hospital General Universitario Gregorio Marañón, 28007 Madrid, Spain; judith.abarca@iisgm.com (J.A.-Z.); mmbarbero@salud.madrid.org (M.L.M.-B.); sara.salvador@iisgm.com (S.S.-M.); maria.sanjurjo@salud.madrid.org (M.S.-S.); 2 Servicio de Neurología, Instituto de Investigación Sanitaria Gregorio Marañón, Hospital General Universitario Gregorio Marañón, 28007 Madrid, Spain; alozanoros87@gmail.com (A.L.R.); mluisa.martinez@salud.madrid.org (M.L.M.-G.); 3 Unidad de Enfermedad Inflamatoria Intestinal, Instituto de Investigación Sanitaria Gregorio Marañón, Hospital General Universitario Gregorio Marañón, 28007 Madrid, Spain; ignacio.marin@salud.madrid.org (I.M.-J.); beatriz.lopez@iisgm.com (B.L.-C.)

**Keywords:** TGF-β signaling, Th17 response, multiple sclerosis, inflammatory bowel disease

## Abstract

The Th17 immune response plays a key role in autoimmune diseases such as multiple sclerosis (MS) and inflammatory bowel disease (IBD). Expression of Th17-related genes in inflamed tissues has been reported in autoimmune diseases. However, values are frequently obtained using invasive methods. We aimed to identify biomarkers of MS in an accessible sample, such as blood, by quantifying the relative expression of 91 Th17-related genes in CD4+ T lymphocytes from patients with MS during a relapse or during a remitting phase. We also compared our findings with those of healthy controls. After confirmation in a validation cohort, expression of *SMAD7* and *S1PR1* mRNAs was decreased in remitting disease (–2.3-fold and –1.3-fold, respectively) and relapsing disease (–2.2-fold and –1.3-fold, respectively). No differential expression was observed for other SMAD7-related genes, namely, *SMAD2*, *SMAD3*, and *SMAD4*. Under-regulation of *SMAD7* and *S1PR1* was also observed in another autoimmune disease, Crohn’s disease (CD) (−4.6-fold, -1.6-fold, respectively), suggesting the presence of common markers for autoimmune diseases. In addition, expression of *TNF, SMAD2, SMAD3*, and *SMAD4* were also decreased in CD (–2.2-fold, –1.4-fold, –1.6-fold, and –1.6-fold, respectively). Our study suggests that expression of *SMAD7* and *S1PR1* mRNA in blood samples are markers for MS and CD, and *TNF, SMAD2, SMAD3*, and *SMAD4* for CD. These genes could prove useful as markers of autoimmune diseases, thus obviating the need for invasive methods.

## 1. Introduction

Human T helper 17 (Th17) cells, together with Th1 cells, play a role in the immunopathogenesis of several human inflammatory diseases, such as multiple sclerosis (MS), Crohn’s disease (CD), rheumatoid arthritis, and psoriasis [1,2,3]. Th17 cells are considered proinflammatory cells and produce cytokines including IL17-A, IL17-F, TNF, IL-22, IL-6, and IL-21 [4]. The TGF-β pathway is a major regulator of T-cell differentiation that governs the Th1/Th17 decision in autoimmune inflammation of the central nervous system (CNS). TGF-β–mediated Th17 differentiation is transmitted via SMAD-dependent or non-SMAD pathways [5,6]. *SMAD7* negatively regulates phosphorylation of the SMAD2/SMAD3 complex, which is necessary for TGF-β signaling [7,8]. Thus, TGF-β, along with other proinflammatory cytokines, such as IL-1β, IL-6, and IL-23 are inducers of human Th17 differentiation [9]. However, TGF-β is considered an anti-inflammatory factor, and the role of TGF-β in the differentiation of Th17 cells remains unclear. In this sense, various roles have been ascribed to subsets of Th17, such as the very pathogenic Th1-like Th17 cells expressing interferon (IFN) [10] and the anti-inflammatory regulatory Th17 cells [11]. A low TGF-β level supports the generation of inflammatory Th17 cells, while a high level increases the generation of regulatory Th17 cells [12]. Furthermore, it has been suggested that this regulation could be driven by the cytokine CCL2 [13]. 

MS is an autoimmune disease that causes inflammation and neurodegeneration in the CNS. During the course of the disease, patients usually experience acute exacerbations of inflammation (relapses) and periods of stable disease (remission). Th17 cells promote blood-brain barrier disruption, thus altering traffic and inducing chronic inflammation, which in turn leads to the degradation of myelin sheaths and axonal injury [14]. Th17 cells and their pro-inflammatory cytokines are involved in most of the autoimmune disorders affecting the CNS [12,15].

IL-17A is over-expressed in brain lesions in MS patients and in experimental autoimmune encephalomyelitis (EAE), a murine model of MS [16]. Preferential recruitment of pathogenic Th17 expressing IFN and IL-17 through the blood-brain barrier has been shown in EAE [17]. In addition, S1PR1, a regulator of lymphocyte egress from lymphoid organs into systemic circulation has also been associated with EAE due to Th17 activation via IL-6 [18].

Identifying markers of the Th17 response in MS is difficult because the damaged area is not easily accessible. However, compared with healthy donors, MS patients were found to have a higher proportion of Th17 cells among CD4+ T cells and higher serum IL-17 and IL-23 levels in peripheral blood [19]. Our objective was to compare the differential expression of a set of Th17-related genes in CD4+ T lymphocytes between MS patients during relapse and remission and healthy donors. We also aimed to validate the results in CD.

## 2. Results

### 2.1. Patients Characteristics

One hundred subjects were included in the study and distributed in four groups: Remittent recurrent multiple sclerosis (RRMS) during a relapse (*n* = 43), RRMS during a remitting phase (*n* = 21), healthy donors (*n* = 20), and Crohn’s disease (CD) during a relapse (*n* = 16). The patients’ characteristics are shown in Table 1. The main differences between the groups were the higher proportion of men, longer time from diagnosis to sample collection, and the absence of treatment-naïve patients in the CD group. 

### 2.2. StellARrays 

Among 95 Th17-related genes, we found that the only statistically significant differences were observed for expression of *SMAD7*, which decreased by –4.12-fold in RRMS patients during a relapse compared with healthy donors (*p* = 0.023) (Table 2) (see also Table A1 in Appendix A). We decided to test the top three genes. *CSF3* was ruled out because it had very low expression and the melting curve showed the amplification of multiple fragments. Thus, *SMAD7*, *TNF*, and *S1PR1* were selected for further analysis.

### 2.3. Gene Expression of Th17-Selected Genes in MS

**Figure 1:**
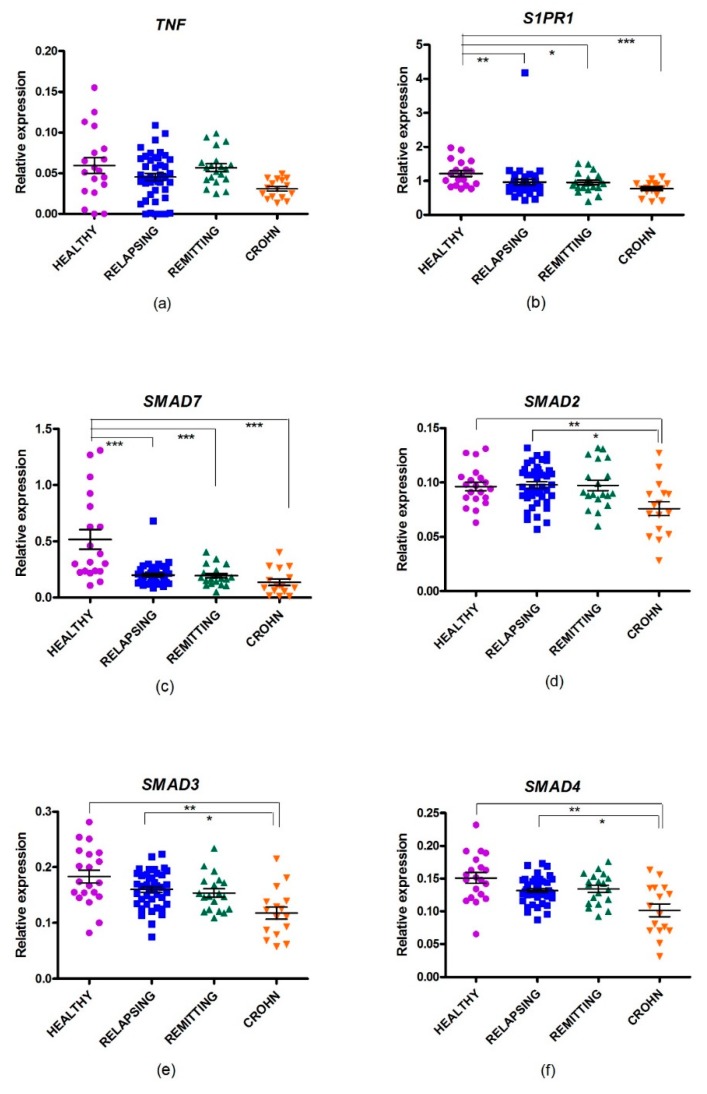
Relative expression of selected genes in multiple sclerosis patients in a relapsing or a remitting phase, in relapsing Crohn’s disease, and in healthy donors. (**a**) *TNF*; (**b**) *S1PR1*; (**c**) *SMAD7*; (**d**) *SMAD2*; (**e**) *SMAD3*; and (**f**) *SMAD4*. * *p* < 0.05; ** *p* < 0.01; *** *p* < 0.001.

Gene expression of the previously selected genes was analyzed in the four groups of individuals (Figure 1, Table 3). Comparison of healthy donors (HD) with the RRMS relapse group showed that mRNA expression of SMAD7 was decreased in the last group (–2.17-fold, PFDR = 0.001), thus confirming the differential expression of SMAD7 between the groups, as previously observed in the StellARrays approach (Figure 1). In addition, the trend toward differential expression of S1PR1 observed in the StellARrays was confirmed, albeit now with a statistically significant result. S1PR1 was expressed –1.31-fold in RRMS patients during a relapse compared with HD (PFDR = 0.010).

Comparison of HD with RRMS remitting patients showed that SMAD7 (–2.29-fold, PFDR = 0.001) and S1PR1 (–1.28-fold, PFDR = 0.041) were decreased in RRMS. However, there were no differences in the expression of the three genes analyzed when remitting RRMS patients were compared with relapsing RRMS patients

### 2.4. Gene Expression of SMAD Genes in MS

We then wanted to assess whether other members of the SMAD pathway were differentially expressed. SMAD7 inhibits phosphorylation of SMAD2 and SMAD3, thus blocking TGF-β signaling. SMAD4 binds to phosphorylated SMAD2/3, and the resulting complex drives gene expression in the nucleus [7]. Changes in expression of *SMAD2*, *SMAD3,* and *SMAD4* were analyzed in RRMS during remission and relapse compared with HD (Figure 1, Table 1). No changes were detected in RRMS patients during either relapse or remission. However, a non–statistically significant trend for weak under-expression of *SMAD4* was observed in RRMS during relapse compared with HD. 

### 2.5. Changes in Gene Expression in CD

All of the genes analyzed were evaluated in relapsing CD patients (Figure 1, Table 3). A population of CD patients during a relapse was not included due to the absence of differential gene expression observed between MS patients in a relapse or in remission. The expression of all of them was decreased when compared with the HD group. SMAD7 was the most repressed gene (−4.65-fold, PFDR = 0.001), followed by S1PR1 (−1.59-fold, PFDR = 0.001), TNF (−2.25-fold, PFDR = 0.001), SMAD3 (−1.64-fold, PFDR = 0.001), SMAD4 (−1.60-fold, PFDR < 0.001), and SMAD2 (−1.40-fold, PFDR = 0.001).

Comparison between MS and CD patients showed that expression of *SMAD7*, *TNF*, *SMAD2*, *SMAD3*, and *SMAD4* was lower in CD patients (Table 3).

## 3. Discussion

The dysregulation of the balance between pro-inflammatory Th17 cells and anti-inflammatory regulatory T (Tregs) cells has an important yet elusive role in many autoimmune diseases. This balance depends on several factors, many of which are still to be elucidated. One such factor is TGF-β, which participates in induction of Th17 and Treg cells. This differential role seems to be tissue-dependent, and TGF-β expression seems to be level-dependent [20]. Measurement of Th17-related genes in local lesions could be of immense interest for identification of new biomarkers in autoimmune diseases. However, in MS, the inaccessibility of the lesions requires an alternative approach. Even in diseases such as CD, with easy, yet invasive, access to biopsies through endoscopies, accessing samples for monitoring the outcome of these parameters is subject to ethical requirements.

In this study, circulating CD4+ T lymphocytes were screened for changes in expression of Th17-related genes. The genes selected were then tested in CD patients to assess whether these changes were common to other autoimmune diseases and could serve as potential biomarkers. This approach has been used successfully in human systemic lupus erythematosus, which is also an autoimmune disease, and in CD4+ T cells differentiating to Th17 in the presence of an inhibitor of Th17 response [21,22].

In our study, *S1PR1* and *SMAD7* mRNA levels were downregulated peripherally in CD4+ T lymphocytes of RRMS patients, both during relapse and in stable disease. These genes were also downregulated in the acute phase of CD.

Sphingosine-1 phosphate (S1P) signaling has an essential role in the regulation of lymphocyte egress from lymphoid nodules to peripheral blood [23]. Lymphocyte egress is dependent upon an S1P gradient between the lymphoid tissue (lower concentration) and plasma (higher concentration). Inhibition of this signaling by an S1P1R agonist, such as fingolimod, has been effective in MS. Fingolimod reduces circulating T lymphocytes (including autoreactive ones), protects from neuroinflammation by blocking the effect of *S1PR1* expression in astrocytes [24], and helps to regulate the blood-brain barrier [25]. In rats, a positron emission tomography imaging study showed how S1PR1 is upregulated in the lumbar spinal cord of EAE rats and associated with glial cell activation and immune cell infiltration [26]. It has been suggested that fingolimod might exert its action via this dual central-peripheral mechanism [27] and also through modulation of the Treg/Th17 cell balance by regulation of the Akt-mTOR and MAPK/ERK pathways [28]. Consistent with our results, in systemic lupus erythematosus, *S1PR1* is expressed less in peripheral blood mononuclear cells (PBMCs) of patients than in healthy controls [21,29,30].

As previously mentioned, *SMAD7* negatively regulates phosphorylation of the SMAD2/SMAD3 complex [7,8]. This inhibition increases expression of IL2, a negative regulator of Th17 differentiation, thus leading to inflammation [31]. Unfortunately, the effect of the decrease of *SMAD7* gene expression on SMAD2/SMAD3 phosphorylation could not be studied in our patients because no protein extract was collected from CD4+T cells. In agreement with the lower *SMAD7* levels in CD4+ T cells from the peripheral blood of MS patients observed in the present work, Zhang et al. found values for the microRNA miR-181, a *SMAD7* inhibitor, to be increased in the same cells of MS patients, thus suggesting an increase in TGF-β-mediated Th17 differentiation [32]. In the same way, Meoli et al. showed that *SMAD7* was downregulated in the CD4+ T lymphocytes of RRMS patients and that TGF-β regulates overexpression of *SMAD7* [33]. Other studies based on PBMCs of MS patients underpin our findings for downregulation of *SMAD7* [34,35]. However, contradictory results were reported by other authors, who found that *SMAD7* was upregulated in the CD4+ T lymphocytes of patients with RRMS during a relapse compared with remitting patients or healthy donors [36]. Our results fully agree with those obtained by Meoli et al. and Zhang et al. and support the downregulation of *SMAD7* in the CD4+ T cells of MS patients. 

The opposite is observed in inflamed tissue. *SMAD7* is overexpressed in brain lesions in EAE mice, in human MS [37], and in the intestine of CD patients [38]. Furthermore, in patients with inflammatory bowel disease and high concentrations of *SMAD7* in the intestine, an antisense RNA has proven successful against *SMAD7* [39,40,41]. The high *S1PR1* and *SMAD7* levels reported in inflammatory lesions in MS and other autoimmune diseases and our finding that those levels were suppressed in peripheral blood, points to enrichment of highly expressed *SMAD7* cells at inflammation sites and, in parallel, enrichment of poorly expressed *SMAD7* CD4+ T cells in peripheral blood. In addition, the recently described association of the intestinal *Smad7* expression with multiple sclerosis in a murine model suggests a relevant role of SMAD7 in these autoimmune diseases [42].

*SMAD7* expression is induced by *TNF* inhibiting TGF-β signaling [43]. In this regard, we found a trend toward downregulation of TNF in the CD4+ T cells of MS patients and that this was more pronounced in CD patients. The trend was statistically significant when CD patients were compared with MS patients and was consistent with *SMAD7* expression. 

*SMAD7* was the only SMAD gene downregulated in MS. This finding is in agreement with those of a previous study, in which *SMAD2*, *SMAD3*, *SMAD4,* and *SMAD7* were measured in methylprednisolone-treated RRMS patients [44]. However, in CD, we found downregulation of *SMAD2*, *SMAD3*, and *SMAD4*, in addition to *SMAD7*, probably owing to more pronounced downregulation of SMAD transcription in CD than in MS, although this hypothesis requires further investigation.

The consequences of SMAD regulation are not altogether clear, because TGF-β has a dual role in inflammation by promoting anti-inflammatory Tregs, as well as pro-inflammatory Th17 cells [45]. In fact, all T helper subsets have shown this dual pathogenic and protective role. 

Finally, we investigated the expression of Th17 genes. It is known that *IL17F* levels are higher in RRMS patients than in healthy donors and that these levels can discriminate between different phenotypes of MS [46]. However, in our cohorts, no differences were found for *IL17A* or *IL17F*, probably owing to the low expression level observed in our samples. Recently, *IL22*, which also codes for a classical Th17 cytokine, has drawn attention. It is not co-expressed with *IL17A* in the CD4+ T lymphocytes of MS patients [47], and *IL22* mRNA is upregulated in circulating cells of relapsing MS patients compared with remitting patients and healthy donors. Our results for the Th17 gene panel support both findings, albeit without statistical significance. *IL22* mRNA was expressed two-fold higher in RRMS patients during a relapse than in healthy donors. However, this gene was not selected for further analysis and confirmation because of its high *p*-value, suggesting that Th populations other than Th17, such as Th22, could play a role in MS. Consequently, they cannot be ruled out.

The apparent inverse correlation of *SMAD7* and *S1PR1* expression in peripheral CD4+ T cells and at sites of inflammation of patients with immune-mediated diseases could help to obtain information about inflamed sites using a simple blood extraction, a non-invasive method, instead a biopsy. As a limitation of the study, CD patients were more heterogeneous than MS patients in terms of concomitant medication with putative influence in gene expression. In addition, a group with non-active CD was not included because of no differences were observed between relapsing or remitting MS patients.

A regulatory effect of CCL2 in TGF-β regulation and in the balance between Th1/Th17 and Treg in EAE mice has been suggested [13]. However, in our human cohort no differential expression was recorded for *CCL2* or its receptor *CCR2*. Given that the regulatory effect was observed after administering a very low dose of CCL2, it could be interesting to analyze *CCL2* expression in larger cohorts.

In summary, we found that expression of *SMAD7* and *S1PR1* in CD4+ T cells in peripheral blood were biomarkers of MS and CD. In addition, *TNF*, *SMAD2, SMAD3*, and *SMAD4* were downregulated in CD.

## 4. Materials and Methods 

### 4.1. Patients

Samples from patients were provided by the Neurology and Gastroenterology Departments of Hospital General Universitario Gregorio Marañón, Madrid, Spain. Samples were processed immediately upon reception. The inclusion criteria were as follows: a diagnosis of MS or clinically isolated syndrome either during relapse or remission; Crohn’s disease during an acute phase. MS disease activity was defined as any new symptoms or worsening of pre-existing neurologic symptoms lasting more than 24 h after a period of 30 days of improvement or stability in the absence of infection or fever. This study followed the Declaration of Helsinki and was approved by the Gregorio Marañón Hospital ethics committee. All patients signed a written informed consent. The demographic and clinical data collected included sex, age, type of multiple sclerosis, and treatment status (naïve or active treatment). 

### 4.2. Isolation and Culture of CD4+ T cells

**Figure 2:**
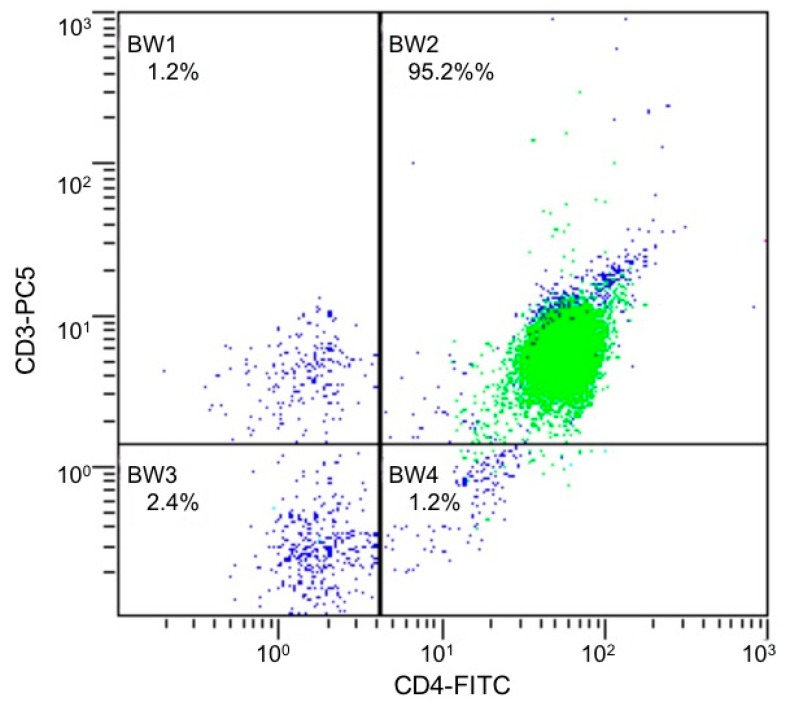
Flow cytometry graph of CD4+ T cells isolated from PBMCs from a representative patient. CD4+/CD3+ represent 95.2%. FICT, fluorescein isothiocyanate; PC5, phycoerythrin-cyanine5. In green, gate of lymphocytes; in blue, total cells.

CD4 T-cells were negatively selected from fresh PBMCs as described by de Andrés et al. [44]. Purity of CD4+ T cells was >95%, as measured by flow cytometry (Figure 2). 

### 4.3. Isolation of RNA and Synthesis of cDNA

RNA was isolated from CD4+ T cells, measured and integrity verified as described [44]. When necessary, RNA was concentrated using Concentrator 5301 (Eppendorf, Hamburg, Germany). Good quality samples (RNA integrity number > 8 were selected. cDNA was generated from 1000 ng (for StellARrays) or 500 ng (for qRT-PCR) of total RNA as described [44]. 

### 4.4. StellARrays Procedure

cDNAs from 6 RRMS patients during a relapse and 6 healthy donors were generated as described elsewhere. Samples were pooled in pairs. Each pair was used to measure the expression of Th17-related genes using a single Human T helper 17 (Th17) 96 StellARray qPCR Array (Bar Harbor Biotechnology, Inc., Trenton, ME, USA). Real-time qRT-PCR was performed using FastStar Universal SYBR Green Master (Roche Applied Science, Penzberg, Germany) in a StepOnePlus system (ThermoFisher Scientific, Waltham, MA, USA) following the StellARrayTM qPCR instructions. Data were analyzed using the Global Pattern Recognition (GPR) algorithm, and the genes used for normalizations were *HS18, NFATC2, CEBPB, STAT4, IL17RA, TRAF3IP2, GATA3, STAT3, TRAF6*, and *NFKB1* [48].

### 4.5. Real Time qRT-PCR

Changes in the selected genes were quantified by qRT-PCR in 20 RRMS patients in remission, 19 healthy donors (HD), 42 RRMS patients during a relapse, and in 16 Crohn’s disease patients during a relapse. Real-time PCR was performed in triplicate using 2 μL/well of a dilution performed for 1/10 of each cDNA (0.04 μM) for *SMAD7, TNF, S1PR1, SMAD2, SMAD3, SMAD4, GAPDH*, and *HPRT1* (*SMAD7* forward, 5′-ACC CGA TGG ATT TTC TCA A-3′; *SMAD7* reverse, 5′-AGG GGC CAG ATA ATT CGT TC’; *TNF* forward, 5′-CAG CCT CTT CTC CTT CCT GAT-3′; *TNF* reverse, 5′-GCC AGA GGG CTG ATT AGA GA-3′; *S1PR1* forward, 5′-AAC TTC GCC CTG CTT GAG-3; *S1PR1* reverse, 5′-TCC AGG CTT TTT GTG TAG CTT-3′; *SMAD2* forward, AAA GGG TGG GGA GCA GAA TA; *SMAD2* reverse, GAA GTT CAA TCC AGC AAG GAG T; *SMAD3* forward, CCA TCC CCG AAA ACA CTA AC; *SMAD3* reverse, TCC ATC TTC ACT CAG GTA GCC; *SMAD4* forward, CCT GTT CAC AAT GAG CTT GC; *SMAD4* reverse, GCA ATG GAA CAC CAA TAC TCA G; *GAPDH* forward, 5′-AGC CAC ATC GCT CAG ACA C-3′, *GAPDH* reverse, 5′-GCC CAA TAC GAC CAA ATC C-3′, *HPRT1* forward, 5′-GAC CAG TCA ACA GGG GAC AT-3′, *HPRT1* reverse, 5′-GTG TCA ATT ATA TCT TCC ACA ATC AAG-3′), 1× SYBR Green PCR Master Mix (Roche Applied Science, Penzberg, Germany) as described [44]. *GAPDH* and *HPRT1* and were used as normalization genes [49]. The results were analyzed using the Relative Quantification app in the Thermo Fisher cloud (Applied Biosystems, Foster City, CA, USA). Relative expression values were represented on graphs using GraphPrism 5.1. The t test was applied for comparisons between groups, and the false discovery rate (FDR) was used to correct multiple testing with a confidence level of 95% and maximum Ct of 35. Efficiency was calculated for each primer pair probe and used for correction.

## 5. Conclusions

Transcription of *SMAD7* and *S1PR1* is decreased in the peripheral blood CD4+ T lymphocytes of RRMS patients during acute relapses and in remitting phases, and in CD patients compared with healthy donors. These genes could prove useful as markers of autoimmune diseases, thus obviating the need for invasive methods.

## Figures and Tables

**Table 1:** Patients characteristics.

Characteristics	RRMS Rel (*n* = 43)	RRMS Rem (*n* = 21)	HD (*n* = 20)	CD (*n* =16)
Median age, years (IQR, range)	35 (14; 22–53)	40 (14.5; 25–52)	29.5 (17.5; 25–61)	28.5 (21.3; 18–69)
Gender Women *n*, (%) Men *n*, (%)	38 (88.4%) 5 (11.6%)	19 (90.5%) 2 (9.5%)	16 (80%) 4 (20%)	7 (43.8%) 9 (56.2%)
Months from diagnosisto sample collection, median (IQR, range)	9 (144; 0–36)	12 (70.5; 0–216)		192 (231; 1–456)
Type of MS/CIS MS *n*, (%) CIS *n*, (%)	33 (76.7%) 10 (23.3%)	13 (61.9%) 8 (38.1%)		
Naïve Yes *n*, (%) No *n*, (%)	24 (55.8%) 19 (44.2%)	16 (76.2%) 5 (23.8%)		0 16 (100%)
Type of treatment in non-naïve (*n*)				
Interferon beta-1a sc	4	2		
Glatitamer Acetate	5	1		
Interferon beta-1b	2	1		
Azathioprine	1			1
Interferon beta-1b im	5			
Methylprednisolone	1			
Dimethyl fumarate	1	1		
Adalimumab				5
Adalimumab + azathioprine				2
Infliximab				1
Vedolizumab				4
Certolizumab + Methylprednisolone				1
Azathioprine + Prednisone				1
Prednisone				1

RRMS, remitting-recurrent multiple sclerosis; rel, relapsing; rem, remitting; HD, healthy donor; CD, Crohn’s disease; MS, multiple sclerosis; CIS, clinically isolated syndrome; mAb, monoclonal antibody; sc, subcutaneous; im, intramuscular.

**Table 2:** Top ten* differentially expressed genes in healthy donors versus RRMS patients with respect to Th17-related genes included in the Human T helper 17 (Th17) 96 StellARray qPCR Array.

Rank	Gene Name	*p*-Value	GPR Fold Change
1	*SMAD7*	0.023116	−4.121045
2	*TNF*	0.097984	−1.975738
3	*CSF3*	0.115770	3.099492
4	*S1PR1*	0.169676	−1.489735
5	*CEBPD*	0.173658	2.447022
6	*IL18R1*	0.181638	1.741877
7	*MMP9*	0.183453	2.454003
8	*ICAM1*	0.204162	−1.397425
9	*IL10*	0.217187	−1.478354
10	*MAP3K14*	0.227840	−1.232434

* Excluded genes used for normalization; GPR, Global Pattern Recognition.

**Table 3:** Statistical analysis and ratios of differential gene expression between groups.

Comparison	*SMAD7* FC (*P_FDR_*)	*S1PR1* FC (*P_FDR_*)	*TNF* FC (*P_FDR_*)	*SMAD2* FC (*P_FDR_*)	*SMAD3* FC (*P_FDR_*)	SMAD4 FC (*P_FDR_*)
HD vs. RRMS rem	−2.29 (0.001)	−1.28 (0.041)	−1.19 (0.405)	−1.28 (0.870)	−1.17 (0.720)	−1.12 (0.155)
HD vs. RRMS rel	−2.17 (0.001)	−1.31 (0.010)	−1.34 (0.123)	−1.05 (0.372)	−1.18 (0.130)	−1.17 (0.110)
HD vs.CD rel	−4.65 (0.001)	−1.59 (0.001)	−2.25 (0.001)	−1.40 (0.001)	−1.64 (0.001)	−1.60 (< 0.001)
RRMS rem vs. RRMS rel	1.05 (1.000)	−1.02 (1.000)	−1.13 (0.690)	1.02 (1.000)	1.01 (1.000)	−1.04 (1.738)
RRMS rem vs. CD rel	−2.02 (0.101)	−1.24 (0.158)	−1.89 (0.003)	−1.3 (0.008)	−1.40 (0.023)	−1.43 (0.004)
RRMS rel vs. CD rel	−2.14 (0.046)	−1.22 (0.137)	−1.67 (0.006)	−1.33 (0.006)	−1.38 (0.016)	−1.40 (0.006)

HD, healthy donor; RRMS, remitting-recurrent multiple sclerosis; CD, Crohn’s disease; rel, relapsing; rem, remitting; PFDR, P value false discovery range.

## Abbreviations

MS	Multiple sclerosis
IBD	Inflammatory bowel disease
CD	Crohn’s disease
Th17	Human T helper 17
CNS	Central nervous system
IFN	Interferon
EAE	Experimental autoimmune encephalomyelitis
RRMS	Remittent recurrent multiple sclerosis
HD	Healthy donors
CIS	Clinically isolated syndrome
mAb	Monoclonal antibody
Tregs	Anti-inflammatory regulatory T
S1PPBMCs	Sphingosine-1 phosphate Peripheral blood mononuclear cells
GPR	Global Pattern Recognition
FDR	False discovery rate

## Appendix A

**Table A1:** Complete differential expression data of genes included in the Human T helper 17 (Th17) 96 StellARray qPCR Array in healthy donors versus RRMS patients.

Rank	Gene Name	*p*-Value	GPR Fold Change
1	*SMAD7*	0.023116	−4.121045
2	*TNF*	0.097984	−1.975738
3	*CSF3*	0.115770	3.099492
4	*S1PR1*	0.169676	−1.489735
5	*CEBPD*	0.173658	2.447022
6	*IL18R1*	0.181638	1.741877
7	*MMP9*	0.183453	2.454003
8	*ICAM1*	0.204162	−1.397425
9	*IL10*	0.217187	−1.478354
10	*MAP3K14*	0.227840	−1.232434
12	*IL15*	0.230006	1.914874
13	*IL12RB2*	0.230664	1.408320
14	*IL17RC*	0.230917	−2.236766
15	*IL22*	0.236646	2.149112
16	*CCR2*	0.236910	1.298827
17	*PTGES2*	0.238409	1.286509
19	*CLEC7A*	0.260678	1.849452
21	*CCR5*	0.280039	1.463742
22	*IL27*	0.280070	2.076678
23	*PRKCQ*	0.280210	−1.203029
24	*PTGS2*	0.287147	−1.292593
25	*CCL5*	0.288848	1.868512
30	*STAT5A*	0.302796	1.180038
31	*TGFB1*	0.303116	−1.169076
32	*IL6R*	0.305101	−1.075965
33	*S100A9*	0.310143	2.053510
36	*ITGAL*	0.312514	−1.163330
37	*IL1B*	0.317977	−1.351223
38	*IL1R1*	0.323904	−1.318258
39	*CD28*	0.326297	−1.154887
40	*S100A8*	0.326948	2.071241
42	*IL17RE*	0.333320	2.006149
43	*IFNG*	0.337986	1.213963
44	*IL17C*	0.342972	1.415405
45	*FAS*	0.348147	1.289762
46	*CD4*	0.354280	−1.157741
47	*IL18*	0.368094	−1.327924
48	*IL12RB1*	0.374193	−1.151614
49	*MAF*	0.380837	−1.055353
50	*LIF*	0.380977	−3.1015170
51	*RORC*	0.385640	1.290307
52	*JAK2*	0.388477	1.082868
53	*IL17D*	0.389396	−1.094554
54	*IL23A*	0.392817	−1.192429
55	*CXCL2*	0.400369	−1.428309
56	*FOXP3*	0.409157	1.087963
57	*ICOS*	0.422018	−1.134658
58	*IL17RB*	0.430393	1.039733
59	*CSF2*	0.433523	1.478325
60	*IL23R*	0.444508	1.133804
61	*EOMES*	0.445174	1.038471
62	*CCR4*	0.446667	1.160488
63	*CCL20*	0.448723	−2.306566
64	*CCR6*	0.468722	1.107297
65	*SYK*	0.471252	1.434643
66	*ITGAX*	0.482659	1.562096
67	*CD8A*	0.482678	−1.206777
68	*SOCS3*	0.484580	−1.111256
69	*LCN2*	0.502556	1.358341
70	*IL21*	0.515282	1.572404
71	*IL2*	0.532927	1.719894
72	*ITGAM*	0.546400	1.047556
73	*FASLG*	0.546997	1.224576
74	*HLX*	0.558206	1.118766
75	*CXCL3*	0.563145	1.062506
76	*MUC5AC*	0.567032	2.128834
77	*IL13*	0.568119	1.404698
78	*IL4*	0.594178	1.031688
79	*CARD9*	0.599793	1.587336
80	*TBX21*	0.615106	1.006864
81	*CXCL10*	0.620610	1.085097
82	*DEFB4*	0.673370	−1.204941
83	*IL17RD*	0.711636	−2.334528
84	*GZMB*	0.713332	1.303447
85	*CCL11*	0.755471	−1.509432
87	*MMP3*	NS	−1.328030
88	*MMP13*	NS	−1.605307
89	*CCL13*	NS	1.166980
90	*CXCL12*	NS	1.726135
91	*IL17F*	NS	1.173435
92	*IL6*	NS	−1.988051
93	*IL12B*	NS	−1.152891
94	*CRP*	NS	−1.287846
95	*IL17A*	NS	−1.526223
96	*IL25*	NS	1.342714

Genes used for normalization and excluded: HS18, NFATC2, CEBPB, STAT4, IL17RA, TRAF3IP2, GATA3, STAT3, TRAF6, NFKB1, and HS genomic.
